# Pterostilbene Suppresses Ovarian Cancer Growth via Induction of Apoptosis and Blockade of Cell Cycle Progression Involving Inhibition of the STAT3 Pathway

**DOI:** 10.3390/ijms19071983

**Published:** 2018-07-07

**Authors:** Wei Wen, Gina Lowe, Cai M. Roberts, James Finlay, Ernest S. Han, Carlotta A. Glackin, Thanh Hue Dellinger

**Affiliations:** 1 Department of Surgery, Division of Gynecologic Oncology, City of Hope Comprehensive Cancer Center, 1500 E. Duarte Road, Machris 1128, Duarte, CA 91010, USA; wwen@coh.org (W.W.); ehan@coh.org (E.S.H.); 2 Department of Developmental and Stem Cell Biology, Beckman Research Institute, Duarte, CA 91010, USA; contactginalowe@gmail.com (G.L.); cglackin@coh.org (C.A.G.); 3 Department of Obstetrics and Gynecology, Yale University, Yale, CT 06520, USA; cai.roberts@yale.edu; 4 Center for Comparative Medicine, Beckman Research Institute, Duarte, CA 91010, USA; jfinlay@coh.org

**Keywords:** pterostilbene, ovarian cancer, cisplatin, combination, synergy

## Abstract

A growing body of evidence has demonstrated the promising anti-tumor effects of resveratrol in ovarian cancer cells, including its inhibitory effects on STAT3 activation. Nonetheless, the low bioavailability of resveratrol has reduced its attractiveness as a potential anti-cancer treatment. In contrast, pterostilbene, a stilbenoid and resveratrol analog, has demonstrated superior bioavailability, while possessing significant antitumor activity in multiple solid tumors. In this study, the therapeutic potential of pterostilbene was evaluated in ovarian cancer cells. Pterostilbene reduces cell viability in several different ovarian cancer cell lines by suppressing cell cycle progression and inducing apoptosis. Further molecular study has shown that pterostilbene effectively suppressed phosphorylation of STAT3, as well as STAT3 downstream genes that regulate cell cycle and apoptosis, indicating that inhibition of STAT3 pathway may be involved in its anti-tumor activity. The addition of pterostilbene to the commonly used chemotherapy cisplatin demonstrated synergistic antiproliferative activity in several ovarian cancer cell lines. Pterostilbene additionally inhibited cell migration in multiple ovarian cancer cell lines. The above results suggest that pterostilbene facilitates significant anti-tumor activity in ovarian cancer via anti-proliferative and pro-apoptotic mechanisms, possibly via downregulation of JAK/STAT3 pathway. Pterostilbene thus presents as an attractive non-toxic alternative for potential adjuvant or maintenance chemotherapy in ovarian cancer.

## 1. Introduction

Epithelial ovarian cancer (EOC) is the most fatal gynecologic cancer among women in the United States [1], with dismal outcomes for advanced stage and recurrent disease. Novel therapies are desperately needed, and this has led to investigation of the JAK2/STAT3 pathways, which appear to be play an important role in carcinogenesis of numerous cancers [2,3,4,5]. This pathway represents an attractive therapeutic target given its tight regulatory steps at multiple cellular levels, thus offering ample targeting points [6,7]. A number of existing drugs and natural compounds inhibit or modulate the JAK/STAT3 pathways, including resveratrol [8,9] and ruxolitinib, an FDA approved drug for the treatment of myelofibrosis (MF), post-polycythemia vera myelofibrosis (PPV-MF), and post-essential thrombocythemia myelofibrosis [10,11]. Ruxolitinib additionally is currently tested in newly diagnosed ovarian cancer in upfront treatment in a clinical trial led by the Gynecologic Oncology Group. Resveratrol on the other hand, has been widely researched, and despite promising preclinical data, has demonstrated disappointing clinical efficacy in patients due to its poor bioavailability [12,13,14]. In contrast, pterostilbene (PTE), a natural, dimethylated resveratrol analog with higher bioavailability, has recently become of increasing interest [15,16,17,18]. Its strong antitumor activity, low toxicity, and commercial availability as well as its low cost, has rendered PTE as an attractive agent for adjuvant therapy in malignancies [19,20,21,22,23,24,25,26,27]. As healthcare costs rise, interest in natural supplements with strong anti-tumor properties has increased, and pterostilbene represents an appealing novel therapeutic agent in chronic cancers such as ovarian cancer, where recurrent disease is common, and long-term goals include maintenance of quality of life along with longevity. We thus evaluated the mechanistic role of pterostilbene in ovarian cancer cells, to test its effectiveness as single agent or adjuvant agent for epithelial ovarian cancer (EOC). We explored its potential role in JAK/STAT signaling, given previous reports in other cancers, including osteosarcoma, where pterostilbene reduced tumor cell adhesion, migration and mitochondrial membrane potential (MMP), and directly inhibited the phosphorylation of JAK2 at Tyr 1007 and the downstream activation of STAT3 [28].

## 2. Results

### 2.1. Pterostilbene Inhibits Ovarian Cancer Cell Growth

**Figure 1:**
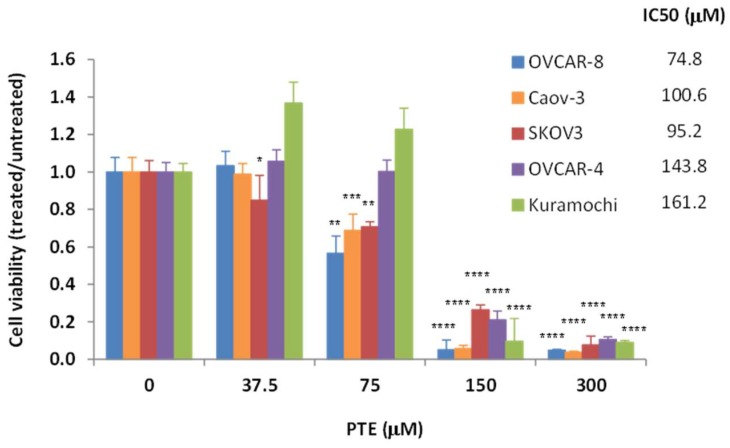
Pterostilbene (PTE) inhibits ovarian cancer cell viability. Cells were treated with vehicle (DMSO) or PTE (37.5–300 μm) for 72 h. Cell viability was determined using MTT assay. Data are expressed as the ratio to control treated with vehicle (DMSO). IC_50_ values of pterostilbene were determined by the Chou-Talalay method. Results are representative of 3 or more experiments, each with 3 or more replicates. *, *p* < 0.05, **, *p* < 0.005, ***, *p* < 0.0005, ****, *p* < 0.0001, versus control treated with vehicle.

To study the anti-tumor activity of pterostilbene in ovarian cancer, we tested its effect on cell growth in several ovarian cancer cell lines, OVCAR-4, OVCAR-8, SKOV3, Caov-3 and Kuramochi. Exponentially growing cells were treated with increasing concentrations of pterostilbene (37.5–300 μm) for 48 h. As shown in Figure 1, pterostilbene significantly reduced cell viability in a dose-dependent manner, with IC_50_ (concentration for 50% growth inhibition) between 75–161 μm. These results indicate that pterostilbene can potently inhibit ovarian cancer cell growth.

### 2.2. Pterostilbene Suppresses Ovarian Cancer Cell Cycle Progression

**Figure 2:**
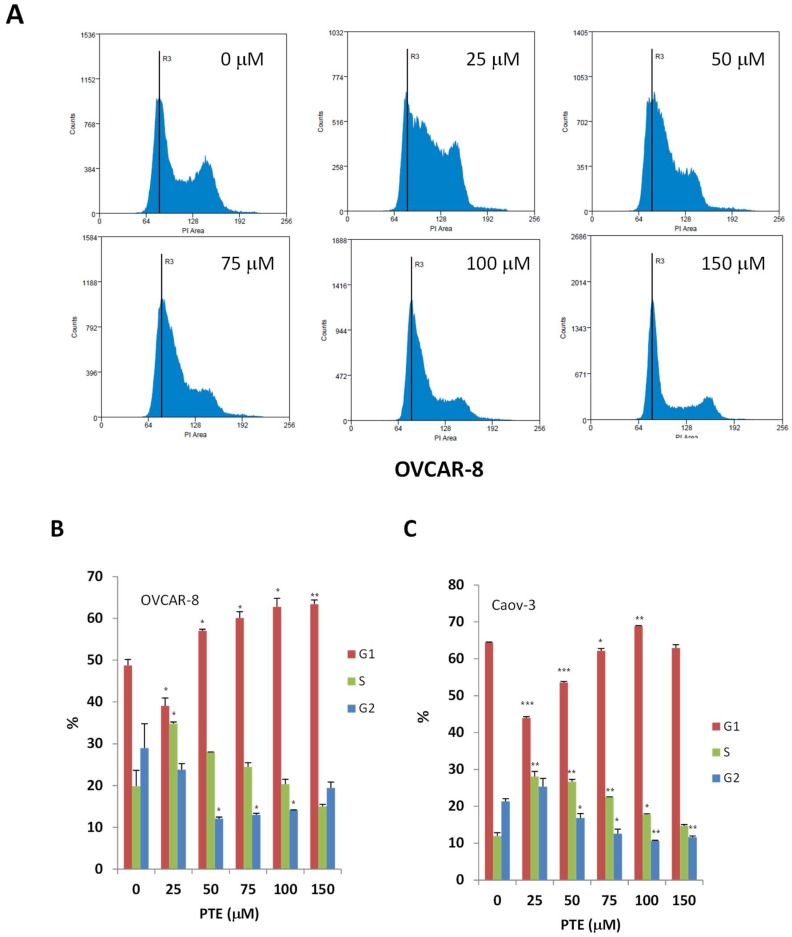
Pterostilbene suppresses cell cycle progression. OVCAR-8 and Caov-3 Cells were treated with vehicle and PTE (25–150 μm) for 24 h. The treated cells were labeled with PI for DNA contents and analyzed by flow cytometry. (**A**) Representative histograms of cell cycle analysis of OVCAR-8. (**B**,**C**) Cell cycle distribution of OVCAR-8 and Caov-3. The data indicate the percentage of cells in each phase of cell cycle. Results are representative of 3 or more preparations. *, *p* < 0.05, **, *p* < 0.005, ***, *p* < 0.0005, versus control treated with vehicle.

We next investigated whether the reduced cell viability was due to inhibition of cell cycle progression. Sub-confluent cells were treated with various concentrations of pterostilbene for 24 h, cells were then labeled with propidium iodide (PI) for DNA content and analyzed by flow cytometry. As shown in Figure 2, the effect of pterostilbene on cell cycle progression appeared to be concentration dependent in both OVCAR-8 and Caov-3 cells. Low concentration of pterostilbene (25 μm) caused an increase of cells in S-phase and a corresponding decrease of cells in G1. With an increasing concentration of pterostilbene, the number of cells entering G1 phase was increasing and the number of cells entering S or G2/M phase was decreasing. These results suggested that pterostilbene might arrest ovarian cancer cells at S phase at low concentration and at G1 phase at higher concentration.

### 2.3. Pterostilbene Induces Ovarian Cancer Cell Apoptosis

**Figure 3:**
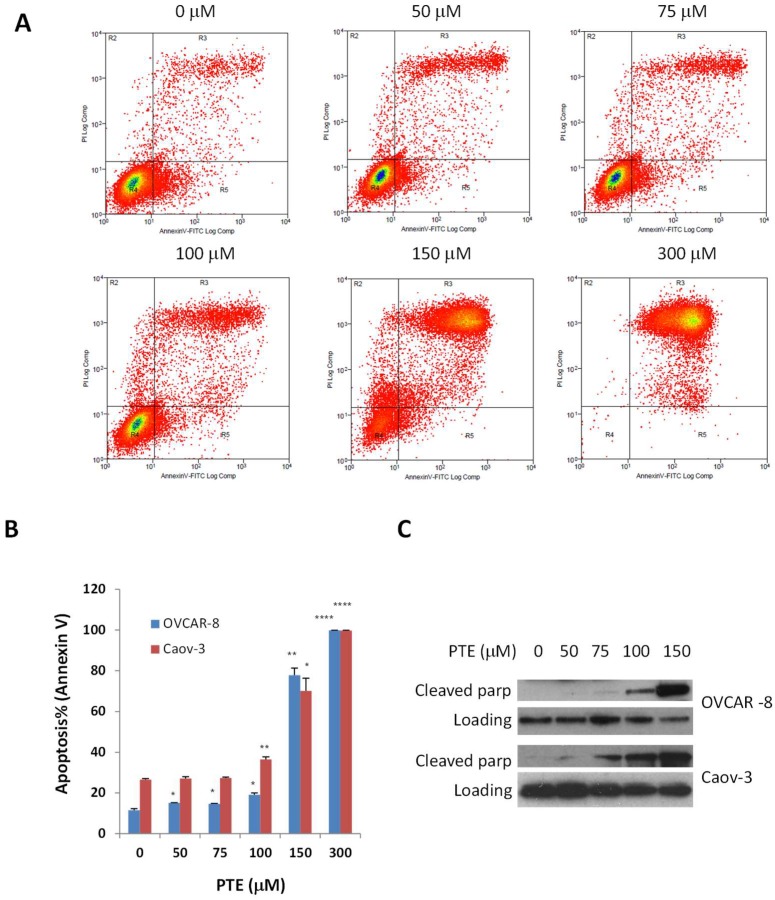
Pterostilbene induces cell apoptosis. OVCAR-8 and Caov-3 cells were treated with vehicle and PTE (25–300 μm) for 48 h. Apoptosis was determined by flow cytometry using annexin V and PI staining (**A**,**B**) or by Western blot for the expression of cleaved poly-ADP ribose polymerase (PARP) (**C**). Results are representative of 3 or more preparations. *, *p* < 0.05, **, *p* < 0.005, ****, *p* < 0.0001, versus control treated with vehicle.

The reduced cell survival by pterostilbene could also be due to the induction of apoptosis. To study this possibility, cells were treated with various concentrations of pterostilbene for 48 h. The number of apoptotic cells was then determined by annexin V staining. As shown in Figure 3, pterostilbene induced cell apoptosis in a dose dependent manner in both OVCAR-8 and Caov-3 cells. After incubation with 50, 75, 100, 150 and 300 μm pterostilbene, apoptotic OVCAR-8 cells increased from 11.5 to 15.1, 14.6, 19.1, 77.9 and 99.8, respectively and apoptotic Caov-3 cells increased from 26.5 to 27.1, 27.3, 36.5, 70.2 and 99.7, respectively. Consistent with the annexin V staining results, more cleaved poly-ADP ribose polymerase (PARP) were generated in both OVCAR-8 and Caov-3 cells treated with pterostilbene for 48 h. PARP is 116kDA protein mainly involved in DNA repair and cell survival. The cleavage of this protein by caspases during apoptosis is considered to be a marker for apoptosis. These results indicate that pterostilbene could effectively inhibit cell viability of human ovarian cancer cells by promoting apoptosis.

### 2.4. Pterostilbene Inhibits Ovarian Cancer Cell Migration

**Figure 4:**
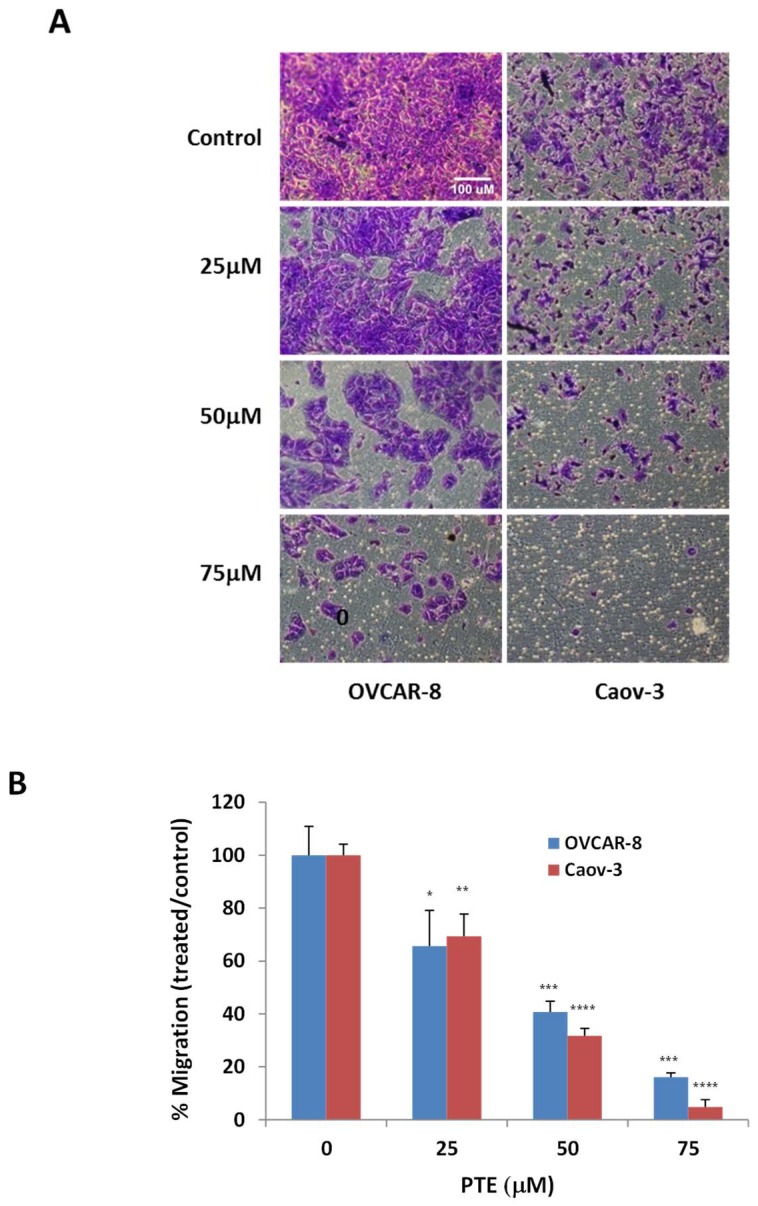
Pterostilbene inhibits cell migration. OVCAR-8 and Caov-3 cells were placed in the upper chamber of a transwell in the presence of various concentrations of PTE. Migrated cells were quantified 2 days after incubation. (**A**) Representative fields of migration for OVCAR-8 and Caov-3 cells. (**B**) The quantification of migration. Data were expressed as a ratio to vehicle control. Results are representative of 3 or more experiments, each with 3 or more replicates. *, *p* < 0.05, **, *p* < 0.005, ***, *p* < 0.0005, ****, *p* < 0.0001, versus control treated with vehicle.

To further understand anti-tumor activity of pterostilbene in ovarian cancer, we studied the effect of pterostilbene on cell migration and invasion using a trans-well assay. OVCAR-8 and Caov-3 cells were incubated with various concentrations of pterostilbene for 48 h. As shown in Figure 4, the number of cells migrating through pores was significantly decreased by pterostilbene in a dose dependent manner in both OVCAR-8 and Caov-3 cells, suggesting pterostilbene could also affect ovarian cancer cell migration. 

### 2.5. Pterostilbene Inhibits STAT3 Signaling Pathway

**Figure 5:**
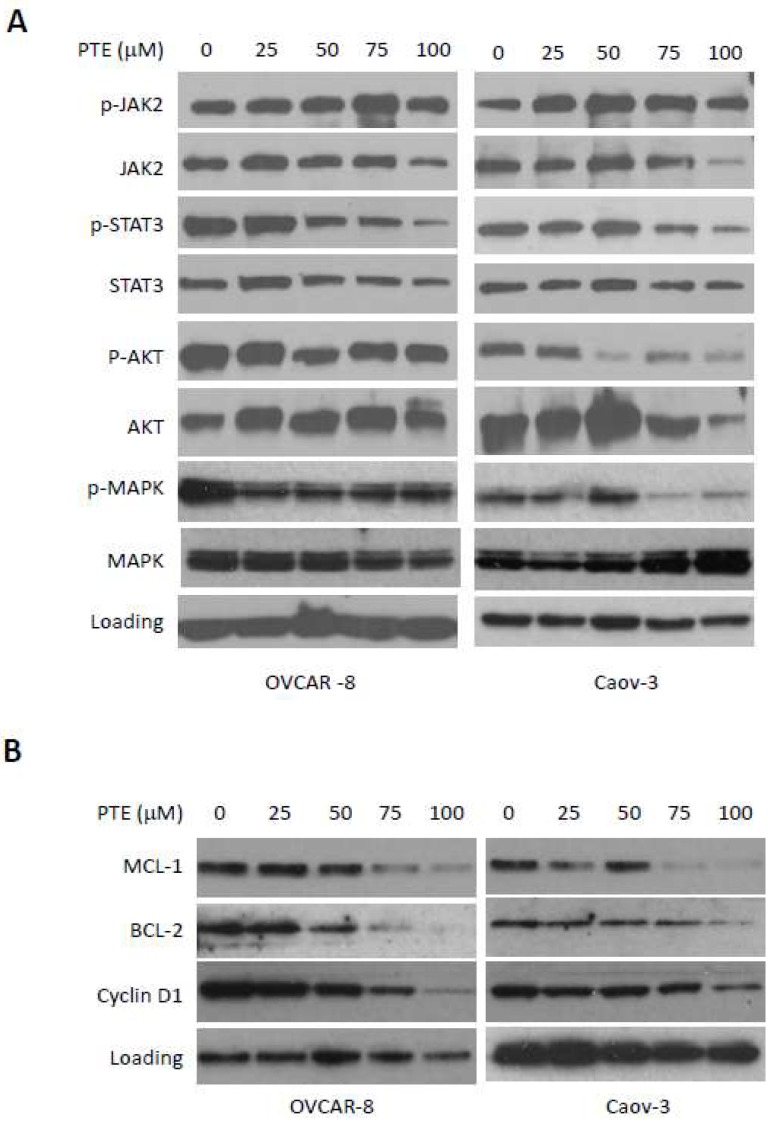
Pterostilbene inhibits STAT3 activation. OVCAR-8 and Caov-3 cells were treated with PTE at various concentrations for 24 h. Cells were harvested and analyzed (**A**) for the phosphorylation of STAT3, AKT and ERK and (**B**) for the expression of STAT3 downstream molecules, MCL-1, BCL-2 and cyclin D1. Results are representative of 3 or more preparations.

To understand molecular mechanism of anti-tumor activity of pterostilbene, we investigated the molecular changes in ovarian cancer cells in response to pterostilbene treatment. A number of signaling pathways, including JAK/STAT3, AKT and ERK pathways, are constitutively activated and play important roles in the growth and progression of human ovarian cancers [29,30]. To study the effect of pterostilbene on these signaling pathways, OVCAR-8 and Caov-3 cells were treated with pterostilbene at various concentrations for 24 h and examined for the expression of p-STAT3, p-AKT and p-ERK. As shown in Figure 5A, pterostilbene treatment caused a reduction of p-STAT3 in both cell lines in a dose dependent manner. However, the inhibition of p-AKT or p-ERK by pterostilbene was only found in Caov-3 cells, but not in OVCAR-8 cells.

We next investigated the effect of pterostilbene on expression of downstream signal molecules of STAT3 activation. Activated STAT3 induces the expression of anti-apoptotic proteins, such as MCL-1, BCL-2 and cell cycle protein, such as cyclin D1. To study the effect of pterostilbene on the expression of these proteins, cells were treated with increasing concentration of pterostilbene for 24 h and studied for the expression of these proteins by Western blot. As shown in Figure 5B, the expression of MCL-1, BCL-2 and cyclin D1 were significantly decreased by pterostilbene in a dose dependent manner, consistent with inhibition of p-STAT3.

### 2.6. Pterostilbene Enhances Anti-Tumor Activity of Cisplatin

**Figure 6:**
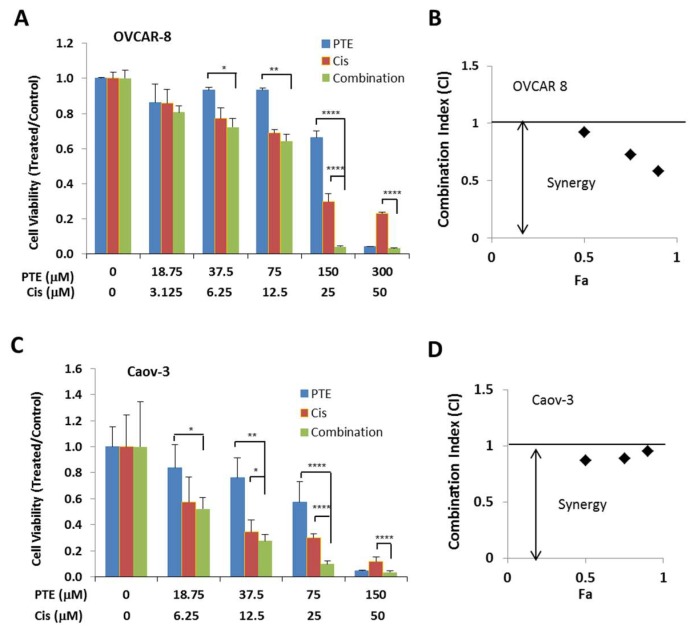
Pterostilbene enhances anti-tumor activity of cisplatin. OVCAR-8 (**A**,**B**) and Caov-3 (**C**,**D**) cells were treated with cisplatin and PTE either alone or in a combination at various concentrations in a fixed molar ratio. Cell viability was determined 48 h later (**A**,**C**). Data are expressed as the ratio to control treated with vehicle (DMSO). (**B**,**D**) Combination Index (CI) values. CI values in black diamond were determined by Chou-Talalay method using the Calcusyn software.Results are representative of 3 or more experiments, each with 3 or more replicates. *, *p* < 0.05, **, *p* < 0.005, ****, *p* < 0.0001, versus control treated with vehicle.

As a widely used first line chemotherapy therapy agent for ovarian cancer patients, cisplatin also causes side effects and drug resistance [3,31]. We tested whether pterostilbene could increase the anti-tumor activity of cisplatin. Both OVCAR-8 and Caov-3 cells were treated with pterostilbene and cisplatin either alone or in combination. The viable cells were determined by MTT assay. As shown in Figure 6, pterostilbene synergistically enhances anti-tumor activity of cisplatin in both OVCAR-8 and Caov-3 cells.

## 3. Discussion

In this study, we investigated the anti-tumor activity of pterostilbene in human ovarian cancer. Our results demonstrated that pterostilbene can inhibit cell viability through suppressing cell cycle progression and inducing apoptosis. We further characterized the mechanism underlying this activity and found that inhibition of STAT3 pathway may be involved in its anti-tumor activity. Pterostilbene also was found to potentiate anti-tumor activity of cisplatin, a first line agent for ovarian cancer treatment. Taken together, our results suggest the potential of pterostilbene for the clinical application in the prevention and treatment of ovarian cancer.

STAT3 is constitutively activated in ovarian cancer and its continued activation is associated with tumor progression and poor prognosis in ovarian cancer patients [5,6,7,32]. It has been previously demonstrated that inhibition of STAT3 pathway effectively suppresses ovarian cancer growth and progression [4,33,34,35]. In this study, pterostilbene was found to inhibit STAT3 activation in both OVCAR-8 and Caov-3 cells. Consistent with this result, pterostilbene also caused decreased expression of STAT3 target proteins, including anti-apoptotic proteins, such as MCL-1 and BCL-2 and cell cycle protein, such as cyclin D1. As a result, pterostilbene treatment leads to the induction of apoptosis and inhibition of cell cycle progression. Although further studies are needed to understand the exact molecular pathways underlying the action of pterostilbene, our study provide novel potential applications for pterostilbene as a small molecule inhibitor of STAT3 with anti-tumor activity.

The effect of pterostilbene on cell cycle progression appears to be concentration dependent in both OVCAR-8 and Caov-3 cells. While low concentration of pterostilbene (25 μm) led to an increase of cells in S-phase, higher concentration of pterostilbene (50–150 μm) caused an increase of cells in G0/G1 phase. Pterostilbene induced cell cycle arrest has been shown in a number of other human cancer cells, including AGS human gastric cancer, LNCap human prostate cancer, human lymphoma cells, and HL-60 human acute myeloid leukemia cells [19,24,36,37]. Pterostilbene has been shown to arrest cells in G0/G1 phase of the cell cycle at high concentration (50–100 μm) in these cells, in S-phase at low concentration (20–25 μm) in gasrtic cancer cells and HL-60 cells [24,36]. Similar results have been reported with resveratrol [38,39]. Consistent with these results, one of the critical proteins controlling cell cycle progression, cyclin D1, was remarkably down-regulated by pterostilbene in a dose dependent manner in both OVCAR-8 and Caov-3 cells. Therefore, it’s possible that pterostilbene induced cell cycle arrest through the cyclin-CDK checkpoint.

Platinum is a first line chemotherapy agent for the treatment of ovarian cancer. However, its inevitable acquisition of drug resistance limits its clinical application in the long-term treatment of ovarian cancer [2,31]. The ability of pterostilbene to increase the anti-tumor activity of cisplatin could potentially enable ovarian cancer cells to response to cisplatin more effectively with decreased toxicity and resistance. More study is needed to explore the clinical potential of this combination therapy and to understand the mechanism underlying the synergy.

In summary, our results demonstrate that pterostilbene, a resveratrol analog with improved bioavailability, reduces proliferation and migration in ovarian cancer cells involving inhibition of the STAT3 pathway, and potentiates the anti-proliferative effects of cisplatin. Pterostilbene thus presents as a potential attractive non-toxic alternative for future study of potential adjuvant or maintenance chemotherapy in ovarian cancer.

## 4. Methods and Materials

### 4.1. Reagents

Pterostilbene (PTE) was kindly provided by Chromadex, Inc., Irvine, CA, USA. Antibodies against p-STAT3 (Y705), STAT3, p-ERK (T202/Y204), ERK, p-AKT (S473), p-JAK2 (Y1007/1008), JAK2, BCL-2, Cyclin D1, PARP, and GAPDH were obtained from Cell Signaling Technology (Danvers, MA, USA). The antibodies against AKT and MCL-1 were from Santa Cruz Biotechnology (Dallas, TX, USA).

### 4.2. Cell Culture

The human ovarian cancer cell line SKOV3 and Caov-3 were from American Type Culture Collection (ATCC, Manassas, VA, USA) OVCAR-4 and OVCAR-8 were from National Cancer Institute and Kuramochi ovarian cancer cell line was obtained from National Institute of Biomedical Innovation JCRB Cell Bank. SKOV3 and Caov-3 cells were cultured in DMEM medium, OVCAR-4, OVCAR-8 and Kuramochi cells were cultured in RPMI1640 medium. Culture media contain 10% FBS and 1% penicillin/streptomycin (P/S). All cells were grown in 5% (*v*/*v*) CO_2_ at 37 °C.

### 4.3. Cell Viability Assay

Cells (5000 per well) were plated in 96-well plate in 100 μL growth medium. Cells were treated with DMSO or drugs the next day at the indicated concentrations and incubated for an additional 48 h. Viable cells were determined by the MTT assay (Promega, Madison, WI, USA) according to manufacturer’s instruction. Briefly, after treating the cells for 48 h, the media were removed and 110 μL of 3-(4,5-dimethyl-thiazol-2-yl)-2,5-diphenyltetrazolium bromide (MTT) dye (10 μL of 5 mg/mL in PBS with 100 μL of corresponding media) were added to each well and incubated for 4 h. The formazan crystals formed were dissolved in 110 μL dimethyl sulfoxide (DMSO) after removing of the media. Absorbance at 570 nm was determined for each well using SPECTRAmax PLUS^384^ Microplate Spectrophotometer. The IC_50_ was determined using the Calcusyn software (Biosoft, Ferguson, MO, USA).

### 4.4. Cell Cycle Assay

Cells were treated with pterostilbene at various concentrations for 24 h. All cells were harvested, washed with PBS, fixed with cold ethanol (70%), and stained with propidium iodide (10 μg/mL) for DNA content. The staining intensity was analyzed by fluorescence-activated cell sorting (FACS) [40].

### 4.5. Annexin V Staining

Annexin V apoptosis detection kit (BD biosciences, Franklin Lakes, NJ, USA) was used to measure apoptosis. Ovarian cancer cells were treated with pterostilbene at various concentrations for 48 h. All cells, both floating and attached cells, were collected and stained with FITC-Annexin V and PI (propidium iodide). The staining intensity was then quantified using fluorescence-activated cell sorting (FACS) [40].

### 4.6. In Vitro Migration Assay

Cell migration was assayed using 24 mm diameter chambers with 8 μm pore filters (BD BioCoat 24-well plate 8.0 micron, BS Biosciences, Franklin Lakes, NJ, USA). Cells were grown in serum-free medium overnight. Cells were trypsinized and resuspended in serum-free medium, and then 200 μL of cell suspension (7.0 × 10^4^ cells) plus pterostilbene (25–100 μm) was added to the upper chambers. The lower chambers were filled with 800 μL of 10% FBS medium and incubated for 2 days for OVCAR-8 cells and Caov-3 cells. The filters were then fixed with 4% PFA for 10 min, washed with PBS 2x and stained with 0.2% crystal violet for 10 min. The upper surfaces of the filters were wiped with Kimwipes^®^ to remove non-migrated cells. The migrated cells were counted using Image-Pro program (Media, Cybernetics, Warrendale, PA, USA).

### 4.7. Western Blot Analysis

Western blots were performed as described previously [27]. Cells were grown in complete medium overnight and treated with DMSO or pterostilbene at various concentrations. The treated cells were washed in cold PBS and lysed in RIPA lysis buffer (Thermo Scientific, Waltham, MA, USA) containing Halt protease and phosphatase inhibitors (Thermo Scientific). The amount of proteins was quantified using BCA protein assay reagent (Thermo Scientific). Equal amount of protein was resolved by SDS-PAGE, transferred to polyvinylidene fluoride membranes, immunoblotted with primary antibody, and detected using a horseradish peroxidase (HRP)-conjugated secondary antibody and chemiluminescent substrates (Thermo Scientific).

### 4.8. Synergistic Analysis

Statistical analysis of synergy was used to evaluate the effect of combined cisplatin/PTE drug treatment. A combination index (CI) for synergy was determined by comparing the anti-proliferative effect of cisplatin or pterostilbene with that of the combination of both drug. This commonly used method was first described by Chou and Talalay [41]. Drug interactions were quantified with the CompuSyn for Windows computer program (MIT, Cambridge, MA, USA). CI values were calculated for the effective doses ED_50_, ED_75_ and ED_90_. A CI < 1 indicates synergy, CI > 1 indicates antagonistic interactions, and a CI value = 1 indicates additive effects [41].

### 4.9. Statistical Analysis

Data are presented as mean ± S.D. Student’s *t*-test was used to compare the means of two groups. All the experiments were repeated 3 times or more. *p* < 0.05 was considered statistically significant.
